# Three-Dimensional Models of Implantation Using Human Stem Cells: Scientific Insights and Broader Considerations

**DOI:** 10.3390/biom15071023

**Published:** 2025-07-16

**Authors:** Megan Munsie, Jock K. Findlay

**Affiliations:** 1 Stem Cell Medicine Theme, Murdoch Children’s Research Institute, Parkville, VIC 3052, Australia; megan.munsie@mcri.edu.au; 2 Melbourne Medical School, University of Melbourne, Parkville, VIC 3052, Australia; 3 Centre for Endocrinology & Reproductive Health, Hudson Institute of Medical Research, Clayton, VIC 3168, Australia

**Keywords:** stem cell-based embryo models, blastoids, implantation, embryo research, regulation, policy, ethics, public engagement

## Abstract

The ability to model the earliest stages of human embryonic development in vitro using pluripotent stem cells offers researchers new ways to understand and interrogate the intricacies of implantation. It also raises important ethical and regulatory considerations, both those common to research involving human embryos, as well as those unique to stem cell-based embryo and endometrial models. This review examines the underpinning scientific discoveries that have led to the development of this rapidly expanding area of research, and how three-dimensional embryo models could be employed in advancing assisted reproductive technologies and understanding implantation failure. Importantly, we also discuss the ethical and legal implications and explore various governance models that have been proposed to foster responsibility and innovation in this area of research. Given the heightened interest in the scientific community on this topic, we finish on the question of how and when to involve the public in the development of this technology and its regulation.

## 1. Introduction

**Figure 1:**
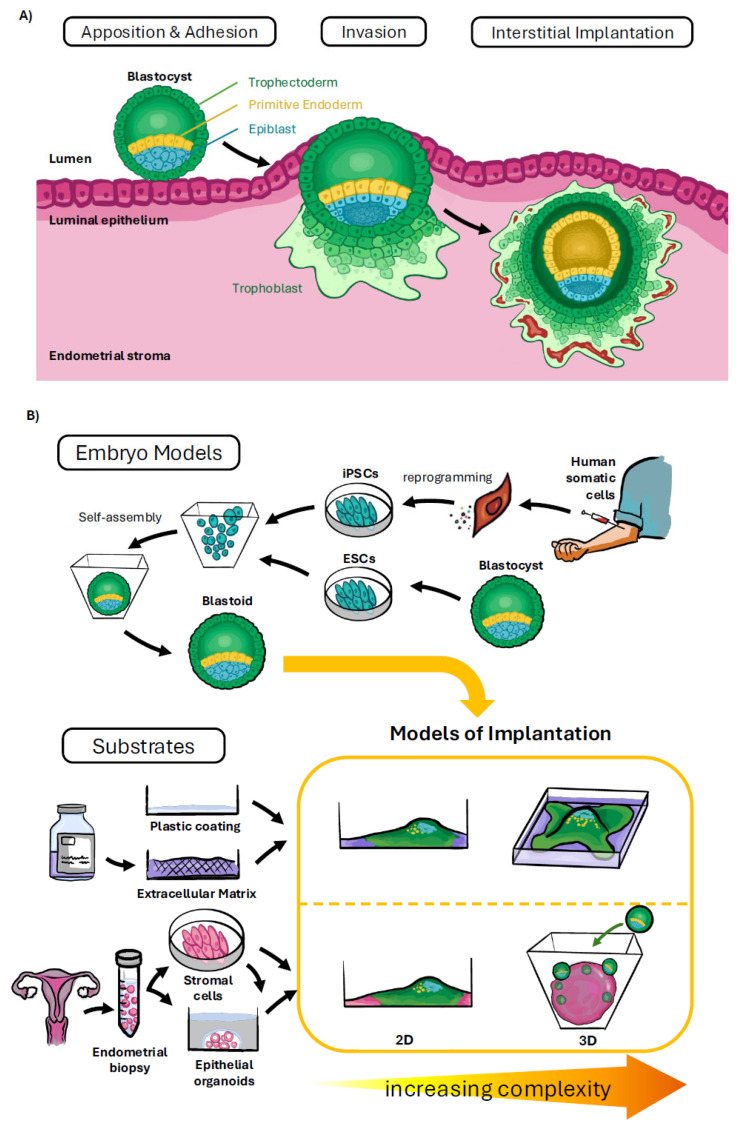
Summary of the different in vitro models of implantation being developed using human stem cells. (**A**) Key steps in implantation; (**B**) in vitro models employed including the sources of cells and substrates.

Researchers have been trying to better understand the “black box” of early pregnancy and the relatively low fecundity in humans for decades [1]. Early studies of the hysterectomy specimens indicated that a high rate of failure appeared to occur during the pre-implantation stage or in the first week following implantation [2]. While further studies have revealed more details about the key events required for the establishment of a human pregnancy—such as apposition and adhesion, invasion, and interstitial implantation (Figure 1A)—questions remain as to why so few embryos are able to attach, penetrate, and invade the endometrial stroma. With up to 40% of pregnancy loss occurring around the time of implantation [1] and the overall success rate of in vitro fertilisation (IVF) procedures remaining less than 30% despite decades of technological advances [3], there is a pressing need for new ways to fully explore this crucial stage of development. Animal models of implantation are of limited value given the distinctive and highly invasive role of the trophoblast in the initiation of implantation in human and non-human primates compared to other species [4]. Improvements to culture conditions have enabled the growth and attachment of human embryos in vitro beyond the blastocyst stage of development [5,6]. However, access to suitable IVF embryos and/or the ability to use human embryos in research can be hampered by regulatory restrictions in certain jurisdictions, making this avenue difficult for researchers to pursue [7]. However, recent advances in stem cell technology may provide an alternative way to study this crucial “black box” stage of development [8,9].

In 2021, two independent research groups described ways to generate three-dimensional (3D) cellular models in vitro from human pluripotent stem cells that mimicked the morphological and cellular composition of human blastocysts derived from fertilisation [10,11]. The models, referred to as “blastoids”, offer researchers the possibility of exploring previously inaccessible aspects of early human embryonic development and implantation. In the short period of time that has followed, protocols have become more efficient in generating stem cell-based embryo models, and researchers have begun to combine these with more complex models of human endometrium that can sustain development in vitro not previously possible [7].

In this review, we discuss the methods used to create human blastoids, and how these have been combined with various matrices and endometrial culture systems to investigate cellular and molecular interactions at implantation. Subsequently, we discuss broader considerations, including proposed governance models, and explore public views on this area of research and the need for ongoing public discourse. Information on the formation and use of endometrial models is more fully covered by several other contributions to this Special Issue of *Biomolecules* [12]. While this review focuses on the role of blastoids in implantation research, a comprehensive description of how human stem cells are used to explore epiblast development and gastrulation post-implantation, or trophoblast and placental development, can be found in the following reviews [13,14].

## 2. Generation of Stem Cell-Derived Embryo Models

The unique capacity of pluripotent stem cells to give rise to cells from all three germ layers in vitro has been the cornerstone of stem cell research for decades. Whether derived from a human blastocyst (referred to as embryonic stem cells or ESCs), or more recently from human somatic cells such as blood or skin cells reprogrammed to a pluripotent state in the laboratory (referred to as induced pluripotent stem cells or iPSCs), researchers have developed increasingly sophisticated methods to coax pluripotent stem cells to self-assemble into 3D structures that reflect key morphological and physiological events during organogenesis. These 3D structures, referred to as organoids, provide new ways to understand and explore developmental biology and congenital disease [15].

There has also been increasing interest in using human pluripotent stem cells to develop 3D models of embryonic development during the peri-implantation and immediate post-implantation period [7]. These models, often referred to as stem cell-based embryo models (or SCBEMs), are broadly categorised and named by the stage of development they represent. For example, models of the blastocyst, referred to as blastoids, have been derived in vitro from pluripotent stem cells and mimic the cellular composition and architecture of the pre-implantation blastocyst 5–7 days after fertilisation including distinct cellular populations characteristic of the epiblast, hypoblast and trophectoderm [10,11,16,17]. This type of SCBEM is described as integrated, as it contains representative cells from both embryonic and extra-embryonic cell lineages (Figure 1B). Other SCBEMs contain representative cells that resemble only some of the cells observed in the post-implantation conceptus between 10 and 14 days after fertilisation. For example, *gastruloids* are 3D cellular clusters that mimic aspects of gastrulation such as germ layer specification and specification of an initial body plan but without the presence of cells representative of extra-embryonic tissues [18].

The potential benefits of research using human SCBEM are wide-ranging, from enhancing our fundamental understanding of the earliest stages of human development to exploring the manifestation of genetic disease during development, as well as discovering new ways to identify embryotoxic drugs and evaluate novel reproductive technologies [7]. However, this review will focus on the possible application of blastoids to enhance our understanding of the key events around human implantation and their implications for research into recurrent pregnancy loss and improving reproductive technologies.

Blastoids were first reported in 2018, when researchers combined mouse embryonic stem cells with stem cells isolated from mouse trophoblast into small aggregates that then self-organised into 3D structures, similarly to mouse blastocysts derived from fertilisation [19]. Subsequent studies showed that blastoids could also be derived from naïve human pluripotent stem cells [10,16,17]. Similar embryo models have also been derived from partially reprogrammed human somatic cells, referred to as iBlastoids [11]. While human blastoids generated in initial studies shared cellular and morphological attributes with human blastocysts derived from sperm–egg fertilisation, the efficiency was initially low and highly variable across the different experiments with between 5.8 and 18% of clusters forming, and some without all pre-implantation lineages represented [10,11]. However, through modifying culture conditions, more recent reports have been able to generate blastoids in a more reproducible manner [20,21] and with an efficiency greater than 70% [16,17]. Importantly, the length of time required in culture to achieve blastoid formation was reduced from weeks to 4 days [22]. While these advances have improved reproducibility, recent analyses of single-cell RNA-sequence data show that current protocols also continue to show transcriptional differences when compared with human blastocysts [23].

## 3. Blastoids and Their Possible Role in Implantation Research

A variety of approaches have been used to explore the capacity of human blastoids to mimic the complex cellular and molecular mechanisms associated with implantation (Table 1). Initial studies placed human blastoids on coated plastic plates where 40–90% attached [10,11]. Multi-nucleation in outer cells was observed to be consistent with a trophoblast-like identity, including cells reminiscent of extravillous cytotrophoblasts and syncytiotrophoblasts, and human chorionic gonadotrophin (hCG) was detected [10,11,16]. Subsequent studies have used co-culture on plates containing a layer of human endometrial epithelial or stromal cells [17,20], implantation and extended culture on 3D extracellular matrices [21,24], and co-culture with 3D endometrial organoids [25,26]. As the surfaces on which the embryo models attach have become more sophisticated, so has the complexity of cellular development.

Two-dimensional culture systems supplemented with endometrial or stromal cells revealed interaction between cellular substrate and embryonic cells replicating key steps in implantation in utero. A third of blastoids were attached when deposited on the hormonally stimulated 2D endometrial stromal culture system containing ciliated and glandular epithelial cells but did not attach to non-stimulated cells [17]. Furthermore, the use of the contraceptive levonorgestrel impaired blastoid attachment [17]. In addition, it was shown that the presence of epiblast-like cells in the blastoids was essential for the maturation of polar trophectoderm-like cells and their ability to interact with endometrial cells, as this ability was lost when simple trophectoderm-only cellular models were compared to blastoids in this culture system [17]. In a similar 2D approach using immortalised primary endometrial stromal cells that were hormonally stimulated, trophoblast proliferation and syncytialisation were induced by blastoids in a similar manner to human blastocysts [20].

In order to progress research into post-implantation development, several groups have further optimised blastoid generation and employed 3D “endometrial” culture systems. A recent approach used thick 3D commercially available extracellular matrices to support complex blastoid-derived structures mimicking embryonic and placental development over 21 days in culture [21]. When the culture system was supplemented with oestradiol, the cellular architecture of the embedded blastoid was more complex than observed using a 2D matrix. They observed a 95% efficiency of attachment via the polar trophectoderm and an expansion of cells representative of invasive extravillous trophoblasts and multinucleated hCG-positive syncytiotrophoblasts. Further culture revealed primary and secondary villous-like projections radiating from the blastoids, consistent with what has been reported previously shortly after implantation in utero. Furthermore, it was observed that epiblast-like precursors appeared to contribute to the trophectoderm before implantation and that the likely source of syncytiotrophoblast cells during the implantation window was the polar trophectoderm [21]. As noted by the authors, further lineage tracing experiments will be required to determine cellular trajectories and the time at which this occurs in vitro compared to in vivo.

Another approach combined blastoids with 3D self-formed endometrial spheroids composed of hormonally stimulated apical endometrial epithelium, dense stromal cells and an endothelial network [26]. Blastoids adhered at a rate around 50%, with hCG-positive syncytiotrophoblast cells appearing around the OCT4+ inner cell mass-like cells, suggesting that the blastoid-derived syncytium interacts with endometrial stromal cells during invasion. The same phenomenon was observed when the experiments were repeated using human-donated IVF-derived blastocysts, albeit at a reduced rate of adhesion [26]. Further studies using this 3D feto-maternal assembloid system could reveal key factors involved in promoting successful implantation [26].

A related recent advance also illustrates the potential for in vitro 3D co-culture systems to help understand implantation failure and endometrial-linked infertility. Researchers showed when primary trophectoderm spheroids were co-cultured with a 3D bilayer of hormonally treated human endometrial and stromal cells that overexpressed a microRNA, miR-124-3p, that is elevated in women with unexplained infertility, there was a block in development in vitro [25]. However, to determine if elevated levels of miR-124-3p affect intact embryos, further studies will be required using blastoids or donated IVF embryos.

Given the increased efficiencies in protocol design, blastoids could also be used to screen for toxicity, optimise media for assisted reproductive technologies, and test novel contraceptives. The observation that the use of the contraceptive levonorgestrel impaired blastoid attachment illustrates the value of this approach [17]. If protocols can continue to be optimised, it may be possible to create large numbers of blastoids and endometrial organoids with distinct and consistent genotypes, perhaps derived from an individual affected by recurrent miscarriage, and explore the feto-maternal interaction. Furthermore, blastoids may identify lineages where gene expression is essential and where it is modified such that the development of the embryo and endometrium is compromised.

While advances in stem cell science have enabled the possibility of using blastoids to gain insights into the intricacies of implantation, and these 3D models may reduce the need to use donated IVF embryos in implantation research, there will still be a requirement to benchmark embryo models against embryos derived from sperm–egg fertilisation. As described above and in Figure 1, the validation of new in vitro implantation models using blastoids is likely to require comparison with donated human sperm–egg embryos.

Although length of time in culture is an obvious and convenient metric, it must be stressed that blastoids and other SCBEM models do not replicate the key morphological and molecular events seen during in utero development in a temporospatial fashion, neither at the anticipated time or context, nor as faithfully organised. It is also important to reiterate that SCBEMs are yet to be shown to be functionally equivalent to human embryos derived from sperm–egg fertilisation. Attempts to achieve pregnancy with blastoids in mice and cynomolgus monkeys demonstrated decidualisation but failed to show the development of a bona fide embryo [19,28]. Indeed, as we will now explore, any attempt to use SCBEMs to achieve a pregnancy following transfer to the womb of a person or any non-human animal is widely condemned.

## 4. Broader Considerations

Alongside scientific advances over the last ten years that have enabled the development of increasingly sophisticated in vitro embryo models, there have been repeated calls to consider the ethical implications of this research and how it should be regulated [29,30,31,32,33,34]. Core to this deliberation has been the question of the moral status of these 3D cellular structures, particularly in relation to existing considerations around human embryo research [33]. This question is particularly poignant when considering the embryo models used in implantation research.

Unlike other SCBEMs, such as gastruloids, which are designed to mimic specific stages of post-implantation human embryonic development, blastoids contain cells representative of all three germ layers, as well as extra-embryonic cell types. As such, blastoids are denoted as being *integrated*, as they model the entire conceptus, and as such, they are deserving of some degree of moral consideration and regulatory oversight [31]. Indeed, in 2021, the International Society for Stem Cell Research (ISSCR), the peak professional body representing stem cell scientists across the globe, recommended that research involving integrated human embryo models should be subjected to review and approval through a specialised scientific and ethical review process, a similar process to that required for the use of human embryos in research [35]. This was in recognition that integrated embryo models could “potentially achieve sufficient complexity to undergo further integrated development” [36]. These guidelines also called for a strict prohibition on any attempts to transfer human SCBEMs to the uterus of a human or an animal host [35]. While these international guidelines provided a valuable framework for the governance of embryo model research, they also acknowledged that existing jurisdictional laws and policies pertaining to the use of human embryos in research also need to be considered. In addition, application of recommendations to specific areas of research, such as reproductive sciences, may require further discussion [37]. Indeed, following feedback from the scientific community, it has been proposed that the ISSCR modify the 2021 Guidelines to remove the delineation between integrated and non-integrated models, and to require all research involving SCBEMs to undergo specialised scientific and ethical review processes [38]. Despite researchers making a careful distinction between research involving human embryos derived from sperm–egg fertilisation and experiments using SCBEMs, how a “human embryo” is defined in law and policies of the jurisdiction where the research will be conducted may impact embryo model research [33,34,39,40].

Although biological definitions might be consistent across scientific communities, legal definitions differ significantly in different countries. Some define a human embryo based on how it was created either limiting it to the act of fertilisation or use of an egg, or by *any* other method, while other jurisdictions refer to developmental potentiality or a combination of these [34,39,40]. For jurisdictions that have introduced regulations and policies pertaining to embryo research, many have also adopted a limit of 14 days after fertilisation as the maximum time permissible for human embryo research to occur. It has been widely debated whether existing embryo research policies and regulation, some introduced more than two decades ago, would also apply to human SCBEM research. A recent analysis identified seven countries—Australia, Austria, Belgium, Germany, Japan, Netherlands and USA—where the legal definition of a human embryo may encompass SCBEMs and thereby impact related research [33]. Ultimately, whether the existing regulations apply to human embryo model research needs to be determined by national regulatory bodies, and it may differ for different types of embryo models [34]. Australian regulators recently made a determination that blastoids met the definition of a human embryo under existing law [41]. Box 1 provides a summary of the decision and impact that this has had on Australian researchers.

Box 1 **Impact of existing human embryo research laws on embryo model research in Australia.**

Since the early 2000s, Australian law^1^ has required research involving human embryos to be licensed by the National Health and Medical Research Council Embryo Research Licensing Committee (ERLC). Following regulatory reforms introduced in 2006, permissible research includes the use of donated excess ART embryos and human embryos arising from processes other than fertilisation, with length of in vitro culture limited to a maximum of 14 days. In June 2020, following the reports by Australian researchers to the ERLC of the development of em-bryo-like structures from reprogrammed somatic cells, an immediate halt was placed on future experiments while the ERLC considered the regulatory implications [11,41]. They determined that this integrated embryo model met the definition of a human embryo under Australian law, and, as such, research would require a licence from the ERLC with standard limits on in vitro development applicable. In October 2022, the ERLC issued the first licence for embryo model research with strict conditions such as specific consent from somatic cell donors for generation of iBlastoids and that a maximum of 117,010 models could be created (Licence 309729) [42]. Furthermore, consistent with limits on human embryo research, experiments would cease prior to 14 days or when the iBlastoid shows evidence of the development of the primitive streak and/or gastrulation [42]. In 2023, the ERLC clarified that embryo models that do not contain extra-embryonic tissues do not meet the definition of a human embryo under Australian law and, as such, did not require a licence at this time [43]. However, the determination also noted that researchers are nonetheless encouraged to seek advice from the ERLC prior to commencing any embryo model research [43]. While Australian law provides a clear regulatory framework for the creation and in vitro culture of human embryo models, arguably along with safeguards for concerned community members, it also imposes restrictions, such as length of culture and accountability for each construct instigated, which are not required in other juris-dictions. In addition, the careful and lengthy deliberation by the regulators over more than two years directly impacted the ability of researchers involved in developing the iBlastoid approach to continue their pioneering research.

^1^ *Research Involving Human Embryos Act 2002 and Prohibition of Human Cloning Act 2002*. 

In addition to guidance provided by ISSCR, several groups in the United Kingdom (UK) have recently proposed possible frameworks to govern human SCBEM research. In July 2024, a group of experts from across biomedical sciences, law, ethics and regulation published the UK Code of Practice for Stem Cell-Based Embryo Models (UK SCBEM Code) [44]. While existing UK law, including the Human Tissue Act 2004 and the Human Fertilisation and Embryology (HFE) Act 1990, has provided a clear framework around human embryo and gamete research for many decades, there was concern about the lack of a clear governance framework for embryo model research in the UK. The UK SCBEM Code was informed by public dialogue [45] and designed to support robust and transparent decision making, and in so doing, encourage public trust. Key features of the UK SCBEM Code include the formation of a dedicated central oversight committee to review all stem cell-based embryo model research on a case-by-case basis, including non-integrated models [44]. No specific limit on the length of time that the models can be kept in culture was set; rather, a requirement for researchers to justify their experimental design to the oversight committee was set, echoing ISSCR recommendations. The UK SCBEM Code also upholds prohibitions on any attempts to transfer models to the womb of a human or animal or extended culture to achieve a viable pregnancy in vitro. Although not legislative, it is proposed that the UK SCBEM Code be widely adopted by UK researchers, research organisations, professional societies, funders and publishers, thereby incentivising researchers to adopt recommendations in order to have their papers published and receive funding [44].

In November 2024, the Nuffield Council on Bioethics published a report and policy brief focused on the ethical and governance questions raised by research involving human embryo models [46]. While they supported the UK SCBEM Code and the immediate formation of a central oversight committee, their recommendation is that in the longer term, human embryo model research should be captured in the HFE Act and a “regulatory sandbox” introduced to test possible approaches to governance for specific types of embryo models without the “burdens of full regulation” [46]. They argue that this staged approach is appropriate given that the science is in its infancy and will limit the risk of disproportionate regulation.

Finally, while the ethical and regulatory considerations pertaining to in vitro models of implantation mainly coalesce around the creation and use of the embryo model, creation of cellular endometrial models will also require compliance to policies and practices around the responsible use of human tissues and appropriate consent from providers, consistent with requirements in other areas of organoid research. What is less clear is what consideration should be given with respect to the donor of the endometrial or stromal cells used to create these increasingly complex embryo/endometrial in vitro models. While it could be anticipated that cell donors who have faced infertility would be supportive of research to better understand implantation failure or improve IVF success rates, the assumption that they would consent to the use of their cells in studies that directly mimic the fusion of an embryo-like blastoid with a sophisticated 3D structure made from their donated cells should be further considered. This should include consideration of whether specific consent should be required for the donor of cells to be used to create the embryo model, as well as the endometrial model. It is worth noting that the terms of the licence recently issued by Australian regulators for blastoid generation require specific consent [42].

## 5. Public Engagement

While the merits of SCBEM research are becoming more recognised by the biomedical scientific community, how—or even if—members of the public are aware of this type of research and its possible benefits is less clear. Separate studies in the Netherlands and the UK have recently explored stakeholder views [45,47].

The Dutch study involved interviews with 27 lay participants who had no prior knowledge of human SCBEM research and were broadly representative of the population, along with seven professionals with experience in developing ethical and legal frameworks for emerging biotechnologies [47]. When asked about their perspective on this area of research, views of both lay and professional Dutch participants ranged from ambivalent to positive or negative. Both cohorts were supportive of SCBEM research on the proviso that the field was regulated. There was also heightened sensitivity around reproductive uses and the involvement of biotechnology companies and commercialisation. Participants also called for a need for the public to be more informed about this area of research and involved in the development of its regulation.

In the UK, online public dialogues were facilitated between 38 members of the public, scientists, legal experts and ethicists [45]. The public participants were recruited from participants of a previously conducted study on early human embryo research. As such, the participants in the UK study already had awareness of embryo models and their context within human embryo research and regulation. Interestingly, when discussing the current status of SCBEM research, views differed, but most saw embryo models as different from human embryos and, as such, did not think the two pose the same moral concerns. The field was seen as an exciting area of research with the potential to improve IVF success rates, understand and reduce the risk of miscarriage and replace human embryos in research. However, participants also worried about stepping into the unknown without “guardrails” [45]. Some participants worried that in the future it may not be possible to differentiate between SCBEMs and embryos derived from sperm–egg fertilisation, and therefore, they wanted to see robust, legislative approaches to governance. Notably, participants were surprised that embryo models were not already regulated and saw the voluntary UK SCBEM Code as a short-term steppingstone to legislation and addressing the “governance gap”. The need for periodic review of the regulatory process to ensure that it keeps up with the science, learns from it, and reflects what society wants was also raised.

While both approaches have provided invaluable initial insights into how SCBEM research and its regulation are viewed, albeit from small, diverse groups, additional studies using similar methodologies in other jurisdictions are required. It would also be interesting to compare the views of those who may benefit from knowledge gained from this form of research, such as people who have suffered recurrent miscarriage or unexplained infertility. Large-scale surveys could provide invaluable insights across populations on specific topics, and Citizen’s Juries, Citizens Assemblies or other deliberative models could be employed where the aim is to reach consensus on recommendations related to policy reforms. Ultimately, the choice of public engagement methods will depend on the purpose, but at this early stage in the development of the technology, all opportunities to facilitate an open exchange of ideas between public participants, scientists, legal and ethical specialists should be encouraged.

Regardless of the public engagement approach taken, a key challenge when engaging public and other stakeholders on this topic is a lack of consistent terminology when referring to these cellular structures. While we have focused on blastoids and introduced SCBEMs in this review, many other names and acronyms are used including embryo-like structures, synthetic embryos, SHEEFs (“synthetic human entities with embryo-like features”), artificial embryos, embryoids and stembryos. While ISSCR and individual researchers call for terms such as “artificial” and “synthetic” to be abandoned [48] and the need to emphasise that these models are not equivalent to IVF embryos when communicating about this area of research to non-expert audiences, media coverage almost always adopts these terms in accompanying headlines. Given that the use of human embryos in research continues to be a divisive issue, perhaps it is less important what the models are called, but more importantly how these 3D structures can contribute to knowledge and advancing ART. SCBEMs hold different scientific value and moral status when compared to embryos originally created for the treatment of infertility and then donated to research when no longer required. Exactly how to achieve this distinction and foster constructive public discourse is an ongoing challenge.

Another consideration when engaging the public in discussion about stem cell embryo models is that this area of research, as raised above, will not replace the need for human embryos and indeed is likely to require the use of human embryos to benchmark observed development and optimisation of future in vitro implantation and developmental models.

Finally, there will need to be ongoing public dialogue around what limits, if any, should be imposed on this area of research as the models become more sophisticated and who should enforce these. This is particularly relevant in jurisdictions where existing embryo research law captures the creation and use of SCBEMs, such as in Australia, where research is permissive but there is a limit in how long the models can be kept in culture, which is not in effect elsewhere in the world. Any change in law to modify regulatory oversight will need to have wide support with the public before policy makers will act. Conversely, reports of non-human SCBEMs able to establish implantation and gestational development in vivo, beyond early implantation/decidualisation already seen, are likely to provide an impetus for greater regulatory oversight from both policy makers and the broader community.

## 6. Conclusions

The use of stem cells has enabled a new avenue for understanding the processes of implantation and related causes of infertility. Rapid progress over less than five years has seen the development of 3D SCBEMs and endometrial models that have the capacity to mimic early implantation up to and beyond gastrulation. Furthermore, such research offers the possibility of using these 3D assembloids to test the effects of toxicity and the potential of new contraceptives.

As laboratories around the world continue to advance understanding of the key events involved in implantation using SCBEMs, ongoing discussions on the ethical implications and appropriate governance models will be required. Initial studies on public attitudes indicate that support for this area of research is likely to be contingent on appropriate regulation. Existing embryo research laws may provide a possible regulatory framework. However, as can be seen in Australia, this may mean that blastoids must be treated as “human embryos” and therefore subject to the same restrictions as research involving donated sperm–egg embryos. New regulatory frameworks such as those suggested by the Cambridge Reproduction and Progress Educational Trust and the Nuffield Bioethics Foundation are to be welcomed. Human stem cell research may be able to shed light on the black box of early development, but this must be carried out in concert with ongoing public discourse on the broader value and possible impacts of this area of research and its regulations.

## Figures and Tables

**Table 1:** Summary of key studies using human stem cell-based models to explore implantation and early human development in vitro.

**Description of Model**	**Observations**	**Significance**	**Reference**
2D attachment of human ART embryos on treated plastic plates	Human blastocysts self-organised to recapitulate many key features of in vivo development up to 12 days post fertilisation (d.p.f.)	Illustrated differences in architecture, cell types and tissue composition between human and mouse development and need for human models to understand human development.	[5]
2D attachment of human ART embryos and human pluripotent stem cell clusters on Matrigel-treated plastic plates	Key morphogenetic events including epiblast and hypoblast segregation; formation of the proamniotic cavity and the bilaminar disc; appearance of yolk sac; and differentiation of the trophoblast into cytotrophoblast and syncytiotrophoblast.	Although not be able to fully recapitulate all aspects of human embryogenesis in vivo, it demonstrated self-organising capacity of human blastocysts.	[6]
3D human blastocyst culture system up to the primitive streak stage of development	Recapitulated timing and lineage segregation and development, more authentically mimicking early human embryonic development in vivo. Revealed molecular and morphogenetic developmental landscape of pre-gastrulation human embryos.	Extended development up to the stage of the primitive streak, providing insights into the pluripotency of stem cells.	[27]
3D blastocyst model (referred to as Blastoid) on treated plastic plates (2D)	Human blastoids resemble human blastocysts in morphology, cell-lineage composition and allocation, and transcriptional state. Recapitulate key morphogenetic events during human peri-implantation development.	First report of human blastoids. Variation in derivation efficiency and cellular composition observed within and between experiments and cell lines. Post-implantation development inefficient.	[10]
3D blastocyst model using reprogrammed adult human fibroblasts (referred to as iBlastoid) on treated plastic plates (2D)	Displayed key features of cellular architecture and molecular markers of pre-implantation human blastocysts including epiblast-, primitive endoderm-, and trophectoderm-like cells; capable of attachment in vitro.	Referred to as iBlastoids as it uses reprogrammed somatic cells rather than pluripotent stem cells as the starting source. Lacked a defined primitive endoderm cell layer.	[11]
3D Blastoid attached on treated plastic plates (2D)	Simple defined culture conditions to generate human blastoids with cellular and molecular fidelity to early human embryos.	Reproducible across different pluripotent stem cell lines and scalable due to simple and efficient approach.	[16]
3D Blastoid attached on hormonally stimulated human endometrial cells derived from organoids (2D)	Increased efficiency in rate of blastoid generation with representation of the three founding lineages (trophectoderm, epiblast and primitive endoderm). Capacity for directionally attachment on hormonally stimulated endometrial cells.	Showed that human blastoids were capable of interacting specifically with hormonally receptive endometrial cells, noting that the contraceptive levonorgestrel impaired blastoid attachment.	[17]
3D Blastoid attached on endometrial stromal cells (2D)	Efficient method for large-scale production of human blastoids with high-fidelity. Blastoid–endometrial stromal cell co-cultures capable of recapitulating maternal–foetal cross talk.	Reproducible using different human embryonic stem cell and induced pluripotent stem cell lines. Showed in vitro attachment of donated human ART blastocysts similar to human blastoids, verifying model.	[20]
3D Blastoids attached on extracellular matrices (3D)	Cellular architecture and molecular markers consistent with early post-implantation development, including expansion and diversification of trophoblast lineages, and robust invasion of extravillous trophoblast cells.	Increased efficiency and optimised conditions with thick 3D matrices enabling more advanced development, offering a continuous and integrated in vitro model system of early embryogenesis.	[21]
3D hormone-responsive human endometrial organoids co-cultured with human blastoids and ART blastocysts	Captured cellular and molecular features of critical implantation stages, including apposition, adhesion, and invasion. This included disruption of endometrial epithelial cells by syncytiotrophoblast cells, which were shown to fuse with endometrial stromal cells.	Model enables visualisation of human embryo–endometrium interaction, suggesting that foetal and maternal cell fusion may occur during early implantation. Described as 3D feto-maternal assembloid.	[26]

## Data Availability

The original contributions presented in this study are included in the article. Further inquiries can be directed to the corresponding author.
